# Preconception Hemoglobin Concentration and Risk of Low Birth Weight and Small-for-Gestational-Age: A Large Prospective Cohort Study in China

**DOI:** 10.3390/nu14020271

**Published:** 2022-01-09

**Authors:** Xiaojing Liu, Hang An, Nan Li, Zhiwen Li, Yali Zhang, Le Zhang, Hongtian Li, Jianmeng Liu, Rongwei Ye

**Affiliations:** 1 Department of Epidemiology and Biostatistics, School of Public Health, Peking University Health Science Center, Beijing 100191, China; liuxiaojing920226@163.com (X.L.); anhang@bjmu.edu.cn (H.A.); zhangyl@bjmu.edu.cn (Y.Z.); zhangle@bjmu.edu.cn (L.Z.); liht@bjmu.edu.cn (H.L.); liujm@pku.edu.cn (J.L.); yerw@bjmu.edu.cn (R.Y.); 2 Institute of Reproductive and Child Health/Ministry of Health Key Laboratory of Reproductive Health, Peking University Health Science Center, Beijing 100191, China

**Keywords:** preconception anemia, low birth weight, small-for-gestational-age, cohort study

## Abstract

Less is known about the impact of maternal preconception anemia on birth outcomes. We aimed to examine associations between preconception hemoglobin (Hb) concentrations with risk of low birth weight (LBW) and small-for-gestational-age (SGA). This study was from a large population-based prospective cohort in China and included 124,725 women with singleton live births delivered at gestational ages of 28–45 weeks who were registered before pregnancy. Maternal Hb concentrations were measured during registration, and other health-related information was recorded prospectively. Logistic regression was used to evaluate the associations between preconception Hb concentrations with risk of LBW and SGA, adjusting for potential confounders. The results showed women with preconception anemia accounted for 22.28%. The incidences of LBW/SGA were 2.37%/6.30% among anemic women, and 2.01%/5.48% among non-anemic women, respectively. Preconception mild anemia increased by 17% (95% confidence interval (CI): 1.06, 1.28) and 14% (95% CI: 1.07, 1.21) the risk for LBW and SGA, while moderate-to-severe anemia had no significant association with LBW and SGA. Compared with the 120–129 g/L group, a U-shaped association was observed between preconception Hb concentrations with LBW and SGA. In conclusion, not only maternal anemia but also elevated Hb concentrations before pregnancy contribute to an increased risk of LBW and SGA.

## 1. Introduction

Anemia is a severe public health problem, especially in developing countries [1]. Over a third of the population drive the anemia burden globally, and females are more likely to suffer from anemia in different regions and at multiple ages [1]. It is estimated that 29% of non-pregnant women are anemic, while the rate rises to 38% after pregnancy as a result of an inequal expansion of the plasma volume and red cells [2]. Maternal anemia, an important indicator of malnutrition status, is considered as a risk factor for various pregnancy complications threatening both the mothers’ and infants’ health [3,4,5].

Low birth weight (LBW), a proxy for assessing intrauterine fetus malnutrition and growth retardation, is associated with neonatal mortality and morbidity, and even long-term health status into adulthood [6,7,8]. Approximately 20 million infants are born with LBW worldwide each year [9]. The notion that maternal anemia during pregnancy relates to LBW has received strong evidential support across epidemiological, medical, and animal studies [10,11,12,13]. A recent systemic meta-analysis found that anemia (hemoglobin (Hb) concentration ≤ 110 g/L) overall or at any time during pregnancy increased the risk of LBW with an adjusted odds ratio: 1.23 (95% CI: 1.06, 1.43) [14]. Compared with the pregnancy period, however, the impact of maternal anemia before pregnancy on LBW remains a key gap in our understanding. From a preventive point of view, understanding the impact of preconception Hb is of particular interest because it is relatively easy and safe to intervene before pregnancy and this may have more effective results on gestational outcomes.

In the present study, small-for-gestational-age (SGA) and birth weight standard deviation score (BWSDS) were also added into our analysis in view of LBW not considering gestational age. We investigated whether anemia prior to pregnancy has any impact on the risk of LBW and SGA with a large historical prospective cohort of reproductive-aged women in rural China. Then, we examined the association between different categories of anemia and Hb concentrations with risk of LBW and SGA at birth.

## 2. Materials and Methods

### 2.1. Background and Original Cohort

In 1993, the Chinese Ministry of Health conducted a large public health campaign to prevent neural tube defects by folic acid supplementation in 21 counties of two southern provinces (Zhejiang and Jiangsu) and one northern province (Hebei) of China [15,16]. Women who were preparing for marriage or who became pregnant were registered in a pregnancy monitoring system that provided principal information on demographic characteristics, prenatal care, and pregnancy outcomes. All women were recommended to supplement 400 µg folic acid daily, starting at the time of registration on the pregnancy monitoring system and continuing until the end of the first trimester. The original cohort included 247,831 women in total, who registered on the monitoring system between October 1993 and delivered by 31 December 1996. The project was approved by the Institutional Review Boards of the United States Centers for Disease Control and Prevention and the Peking University Health Science Center. Informed consent was obtained from all subjects involved in the study.

### 2.2. Selection of Study Subjects

**Figure 1:**
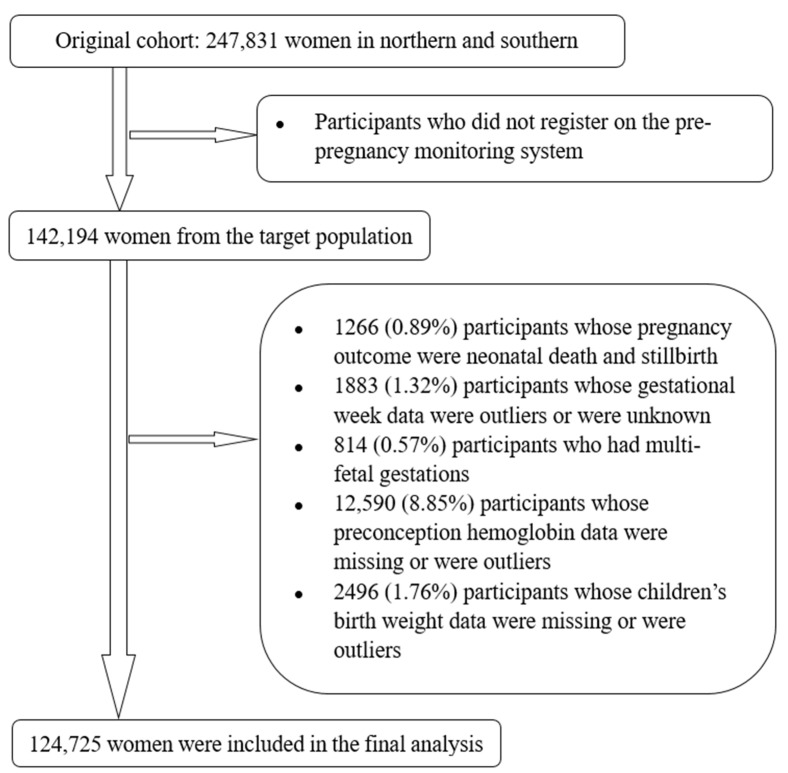
Flowchart of the participants.

To obtain maternal Hb concentrations before pregnancy, we selected women who registered on the pre-pregnancy monitoring system. Of 142,194 targeted participants, we excluded 17,469 women according to the following exclusion criteria: outcomes of neonatal death and stillbirth (*n* = 1266); multiple births (*n* = 814); missing data for preconception Hb concentrations (*n* = 12,585) and Hb concentration < 30 or 200 g/L (*n* = 5); missing data for gestational ages (*n* = 1464) and gestational age < 28 weeks or >45 weeks (*n* = 419); missing data for infant birth weight (*n* = 2183) and birth weight < 1500 g or >6000 g (*n* = 313). Finally, a total of 124,725 women (87.71% of the targeted population) were included in the current analysis. Detailed recruitment criteria of study population arer shown in Figure 1.

### 2.3. Definition of Anemia and Hb Classification

Maternal hemoglobin was measured with a standard cyanmethemoglobin method by using capillary blood via devices available at each hospital; two commonly used devices were the 721 visible spectrophotometer and hemoglobinometer. All project hospitals were provided with standard hemoglobin solutions (50, 100, 150, and 200 g/L) and with a step-by-step procedure for calibrating the hemoglobinometer and for preparing the standard curve for the 721 visible spectrophotometer. To minimize testing bias, in the operating room, step-by-step instructions were placed on the wall to ensure compliance with standard procedures, and equipped with heating devices in winter to maintain the room temperature at over 18 °C. Anemia was defined as Hb concentration below 110 g/L. We further classified anemic women into 2 subgroups (mild anemia: 100 ≤ Hb < 110 g/L; moderate-to-severe anemia: Hb < 100 g/L), and non-anemic women into 4 subgroups by an interval of 10 g/L (110–119, 120–129, 130–139, and ≥140 g/L). The Hb concentrations for the anemia grades are referenced to the World Health Organization (WHO) guidelines [17,18].

### 2.4. Definition of LBW, SGA, and BWSDS

The birth information of infants was extracted from prenatal care and delivery record. Each naked newborn was placed on a digital scale with a new thin clean cloth to measure birth weight with a precision of 10 g. The operation was conducted by trained local health workers within one hour after delivery. All instruments were well calibrated by local authorities of quality and technical supervision. LBW was defined as birth weight of infants < 2500 g. Gestational age was estimated from the first day of the last menstrual period. SGA was defined as gestational-age-adjusted birth weight < the 10th percentile based on the mean birth weight obtained from a national sampling survey in 1998 [19]. BWSDS was calculated based on the mean birth weight and standard deviation of the subjects. We introduced BWSDS into analysis after excluding score limits.

### 2.5. Statistical Analysis

Maternal age and BMI were calculated as mean ± standard deviation (SD), and categorical parameters were expressed as number and proportion. The Student *t*-test was used to compare quantitative variables and the chi-square test for categorical variables between anemic and non-anemic groups. Multiple logistic regression was performed to evaluate the risk ratios (RRs) of LBW and SGA, regression coefficient β, and 95% confidence intervals (95% Cis) by maternal preconception anemia and Hb levels after adjusting for the potential confounders including maternal age, BMI, education, occupation, ethnicity, and folic acid use. All data were analyzed with SPSS v.20.0 software (SPSS Inc., Chicago, IL, USA). The *p* value was calculated by the two-sided test with statistical significance below 0.05.

## 3. Results

Among 124,725 participants in the final study, the incidence of preconception anemia was 22.28%. Baseline characteristics analysis (Table 1) showed women with preconception anemia had a slightly older age, thinner status, and lower education level. In addition, farmer, multiparous, and non-Han ethnicity women were more likely to have suffered from anemia before pregnancy.

The total incidences of LBW and SGA were 2.09% and 5.66%. The incidences of LBW/SGA status were 2.37%/6.30% in women who had preconception anemia, and 2.01%/5.48% in those who did not. Younger age, a lower BMI, lower level of education, occupation as a factory worker, primiparous pregnancy, non-Han ethnicity, and nonuse of folic acid were associated with an increased risk of LBW or SGA status, as well as decreased BWSDS (Table 2). The multiple logistic regression model showed that preconception anemia was associated with 16% (95% CI: 1.02, 1.31) higher risk for LBW, 14% (95% CI: 1.07, 1.20) higher risk for SGA, and a 0.03 SD decrease in BWSDS (95%: −0.04, −0.02) after adjusting for the confounding factors mentioned above (Table 3).

Table 4 shows the associations between preconception Hb concentrations with LBW and SGA. The incidences of LBW/SGA were 2.39%/6.33% and 2.29%/6.18% for women with mild anemia and moderate-to-severe anemia, relative to 2.01%/5.48% for women who were non-anemic. Compared to non-anemia prior to pregnancy, mild anemia significantly increased by 17% (95% CI: 1.06, 1.28) the risk for LBW, 14% (95% CI: 1.07, 1.21) the risk for SGA, and decreased 0.03 SD (95%: −0.05, −0.02) in BWSDS. In the moderate-to-severe anemic group, crude RR of SGA was 1.14 (95% CI: 1.01, 1.28); however, adjusted RR did not show statistical significance in LBW (adjusted RR = 1.10, 95% CI: 0.92, 1.33) nor SGA (adjusted RR = 1.34, 95% CI: 0.99, 1.24). When the 120–129 g/L subgroup was used as the reference, we observed a U-shaped association between maternal Hb levels before pregnancy with LBW, SGA, and BWSDS. Generally, the risk of LBW was higher than SGA in women with abnormal Hb concentrations. In addition, exclusion of major external birth defects from the analysis did not change our results (data not shown).

## 4. Discussion

We performed a large population-based cohort study on the impact of maternal preconception anemia on LBW and SGA at birth. Importantly, our results demonstrated that preconception anemia significantly increased infants in terms of LBW and SGA status, and the effects were evident in women who were mildly anemic prior to pregnancy. We also found a general U-shaped relationship between preconception Hb concentrations and delivering newborns with LBW and SGA; that is, there were a higher risk of LBW and SGA among women with both lower and higher Hb concentrations. The significance of examining the impact of preconception Hb level on LBW and SGA is shown.

Maternal health status around the periconceptional period, when the conceptus is vulnerable to abnormal influences as claimed by the Developmental Origins of Health and Disease (DOHaD), is highlighted due to its great importance in placental function as well as fetus growth and development [20]. So far, multiple studies have identified that reduced Hb levels can suppress placental angiogenesis and transfer less oxygen to the fetus, consequently causing intrauterine growth restriction and LBW [21,22]. However, most studies have focused on the maternal Hb concentrations during the pregnancy period [3,4,23]. As summarized in a recent systemic review and meta-analysis [5], there is an increased risk of LBW among women with lower Hb concentrations during overall or any stage of pregnancy, while elevated Hb concentrations are significantly associated with LBW and SGA only in the second trimester. Evidence pointing to the time before pregnancy is insufficient. A large population-based cohort study conducted in China between 2013 and 2015 [24] demonstrated that pregnant women with Hb < 110 and ≥150 g/L before pregnancy have an obviously increased risk of very preterm birth, which was recognized as a risk factor of LBW [25]. Therefore, we focused on the associations of preconception Hb concentrations with LBW and SGA status at birth in the current study.

### 4.1. Associations between Preconception Hb Concentrations with LBW and SGA

To our knowledge, only two epidemiological studies have examined the association between maternal Hb levels before pregnancy and the risks of LBW and SGA. Ronnenberg et al. performed a study [26] including 405 women and measured their preconception Hb concentrations from a prospective cohort in southern China. The results showed that LBW risk, compared with non-anemia, increased significantly in the presence of moderate anemia (Hb < 95 g/L) (adjusted OR: 6.5, 95%CI: 1.6, 26.7) but not mild anemia (95 ≤ Hb < 120 g/L) (adjusted OR: 2.0, 95%CI: 0.7, 5.9). This estimation was based on a limited sample number and should be interpreted with caution. Additionally, one Korean respective cohort study by Yi et al. [27] verified both preconception mild anemia (100 ≤ Hb < 120 g/L)/moderate-to-serve anemia (Hb < 100 g/L) were associated with an increase of 21%/81% risk of LBW, and 15%/71% risk of SGA, respectively. Their results also indicated that preconception elevated Hb concentrations (Hb ≥ 130 g/L) had no significant effects on LBW and SGA births. These two studies showed similar results to ours in that preconception anemia is related to the delivery of newborns with LBW and SGA. However, our finding of an association between preconception Hb concentration over 140 g/L and significantly increased risk of LBW was contrary to the above studies. The reasons for this inconsistency could be differences in race, study design, or cutoff of Hb concentrations, which should be taken into consideration further for more definite evidence.

In this study, we were surprised to find no significant difference in the risk of LBW or SGA between moderate-to-serve anemic women and non-anemic women. This finding perfectly fits one previous study about preterm birth mentioned above. Considering that knowledge about diseases can influence motivations for behavior change [28], one possible explanation is that slightly abnormal Hb levels may be neglected; however, women with very low Hb levels may automatically promote health care or nutrition supplementary that contribute to an improvement of birth outcomes. Relevant studies are needed in the future. In addition, LBW is recognized to be driven from preterm-birth infants born with small-for-gestational-age at term or growth dysplasia born appropriate-for-gestational-age at term [25,29]. We found that preconception anemia induced a higher risk of LBW than SGA, and elevated Hb concentrations up to over 130 g/L were only associated with a statistically significant risk of LBW (adjusted RR:1.33, 95% CI: 1.08, 1.65) rather than SGA. An explanation for the higher association of abnormal preconception Hb concentrations with LBW than SGA resulted not only from preterm birth but also inadequate growth for appropriate-for-gestational-age birth.

### 4.2. Strengths and Limitations of the Study

The present study used a large population-based prospective cohort to collect information of baseline and birth outcomes, thereby it had great strength to prove associations between them, as well as to minimize recall bias. In addition, we evaluated the risk for LBW and SGA while excluding congenital anomalies and stillbirths, thus reducing information bias. Besides classification by anemia severity, the sample size was large enough to be classified into subgroups by Hb concentrations to gain a comprehensive cause-and-effect relationship. The participants were from southern and northern regions of China, covering a representative sample of the overall Chinese population, which made our findings more reliable and meaningful.

However, several limitations should be addressed when interpreting the results. Since our study analyzed existing data, some potential confounders, such as maternal smoking, air pollution, and the main socioeconomic characteristics of the spouse, were not measured at the time of the original study. Besides, the participants in the current study tended to be vegetarians under a poor economic condition. Maternal diet structure, as a potential confounder, was also not measured at the time. Although adjusting for various socio-demographic factors, we failed to gain information on anemia etiology or relevant variables, such as iron, ferritin, and vitamin concentrations, which has been demonstrated to be associated with adverse birth outcomes [30,31,32]. Finally, SGA was calculated by gestational age, which was based on the last menstrual date and might be miscalculated under the condition of irregular menstruation, resulting in an inaccurate estimation of SGA risk.

## 5. Conclusions

In this study, we found that preconception anemia, besides maternal and socio-demographic factors, was associated with increased risk of LBW and SGA at birth. Higher risks of LBW and SGA were induced by both lower and elevated Hb concentrations. This evidence calls for targeted health surveillance of anemic women before pregnancy to protect against LBW and SGA. Furthermore, anemia etiology, cutoff, and timing of Hb concentrations around periconception are all urgent research agendas for maternal and offspring health. These are also pivotal to propose specific public health policy for reproductive-aged women.

## Figures and Tables

**Table 1:** Characteristics of Chinese women who enrolled in the pregnancy monitoring system according to preconception anemia from 1993–1996.

Characteristics	Anemic Group (*n* = 27,783)		Non-Anemic Group (*n* = 96,942)		*p*
	*n*	%	*n*	%	
Age (years, mean (SD))	24.46 (2.11)		24.35 (2.03)		<0.001
BMI (kg/m^2^, mean (SD))	20.42 (2.18)		20.47 (2.15)		0.001
Education					<0.001
High school or higher	2688	18.42	11,906	81.58	
Junior high school	17,689	21.71	63,771	78.29	
Primary school or Lower, or unknown	7406	25.83	21,265	74.17	
Occupation					<0.001
Farmer	16,108	22.80	54,549	77.20	
Factory worker	10,432	22.07	36,843	77.93	
Other or unknown	1243	18.30	5550	81.70	
Parity					<0.001
Primiparous	26,650	22.09	93,975	77.91	
Multiparous	1133	27.63	2967	72.37	
Ethnicity					0.037
Han	27,544	22.25	96,228	77.75	
Other race	239	25.08	714	74.92	
Folic acid use					<0.001
No	6582	27.69	17,186	72.31	
Yes	21,201	21.00	79,756	79.00	

BMI, body mass index; SD, standard deviation.

**Table 2:** Incidences and crude RR of LBW and SGA according to preconception anemia and other women’s characteristics, China, 1993–1996.

Characteristics	No. of Participants (*n* = 124,725)	LBW (*n* = 2610)			SGA (*n* = 7064)			BWSDS		
		Incidence, %	RR	95%CI	Incidence, %	RR	95%CI	Mean ± SD	ß	95%CI
Age, years										
<20	114	2.63	1.36	0.42, 4.42	2.63	0.63	0.20, 2.01	−0.25 ± 1.04	−0.40	−0.59, −0.21
20–25	86,242	2.07	1.07	0.81, 1.41	5.75	1.42	1.17, 1.72	−0.01 ± 1.00	−0.15	−0.19, −0.12
25–30	35,700	2.15	1.10	0.83, 1.47	5.58	1.38	1.13, 1.67	0.01 ± 1.00	−0.14	−0.18, −0.10
≥30	2669	1.95	1	N/A	4.12	1	N/A	0.15 ± 1.03	0	N/A
BMI, kg/m^2^										
<18.5	19,835	2.94	1.53	1.39, 1.68	7.98	1.56	1.47, 1.66	−0.18 ± 1.00	−0.20	−0.21, −0.18
18.5–23.9	98,190	1.95	1	N/A	5.25	1	N/A	0.02 ± 0.99	0	N/A
24–27.9	6174	1.67	0.85	0.70, 1.04	4.91	0.93	0.83, 1.05	0.22 ± 1.06	0.20	0.17, 0.22
≥28	526	1.52	0.78	0.39, 1.56	3.80	0.71	0.46, 1.12	0.35 ± 1.13	0.33	0.24, 0.41
Education										
High school or higher	14,594	1.81	1	N/A	4.69	1	N/A	0.05 ± 0.99	0	N/A
Junior high school	81,460	2.03	1.12	0.99, 1.28	5.43	1.17	1.07, 1.27	0.01 ± 0.99	−0.04	−0.05, −0.02
Primary school or lower, or unknown	28,671	2.42	1.34	1.17, 1.55	6.82	1.49	1.36, 1.63	−0.05 ± 1.02	−0.10	−0.12, −0.08
Occupation										
Farmer	70,657	2.00	1	N/A	5.54	1	N/A	−0.01 ± 0.99	0	N/A
Factory worker	47,275	2.31	1.16	1.07, 1.26	6.02	1.09	1.04, 1.15	−0.002 ± 1.02	0.004	−0.01, 0.02
Other or unknown	6793	1.60	0.80	0.66, 0.98	4.49	0.80	0.71, 0.90	0.08 ± 1.01	0.09	0.07, 0.12
Parity										
Primiparous	120,625	2.09	1.02	0.82, 1.27	5.70	1.27	1.09, 1.47	−0.01 ± 1.00	−0.15	−0.18, −0.12
Multiparous	4100	2.05	1	N/A	4.56	1	N/A	0.14 ± 1.07	0	N/A
Ethnicity										
Han	123,772	2.08	1	N/A	5.65	1	N/A	0.001 ± 1.00	0	N/A
Other race	953	3.67	1.79	1.28, 2.52	7.35	1.32	1.04, 1.69	−0.16 ± 1.01	−0.16	−0.22, −0.10
Folic acid use										
No	23768	2.28	1	N/A	5.80	1	N/A	−0.02 ± 1.01	0	N/A
Yes	100,957	2.05	0.90	0.81, 0.99	5.63	0.97	0.91, 1.03	0.004 ± 1.00	0.02	0.01, 0.04
Preconception anemia										
No	96,942	2.01	1	N/A	5.48	1	N/A	0.01 ± 0.99	0	N/A
Yes	27,783	2.37	1.18	1.08, 1.29	6.30	1.16	1.10, 1.23	−0.03 ± 1.02	−0.04	−0.05, −0.03

No indicates number; LBW, low birth weight; SGA, small-for-gestational age; RR, risk ratio; CI, confidence interval; BWSDS, birth weight standard deviation score and N/A, not applicable.

**Table 3:** The association of preconception anemia with LBW, SGA, and BWSDS by multiple logistic regression in China, 1993–1996.

Risk Factors	LBW		SGA		BWSDS	
	Adjusted RR	95% CI	Adjusted RR	95% CI	ß	95% CI
Age						
<20	1.27	0.38, 4.12	0.61	0.19, 1.96	−0.40	−0.58, −0.21
20–25	1.00	0.76, 1.33	1.35	1.11, 1.64	−0.13	−0.16, −0.09
25–30	1.04	0.78, 1.38	1.30	1.07, 1.59	−0.11	−0.15, −0.07
≥30	1	N/A	1	N/A	0	N/A
BMI	1.10	1.08, 1.13	1.11	1.09, 1.12	−0.05	−0.06, −0.05
Education						
High school or higher	1	N/A	1	N/A	0	N/A
Junior high school	1.21	1.06, 1.38	1.23	1.13, 1.34	−0.06	−0.08, −0.04
Primary school or lower, or unknown	1.50	1.29, 1.73	1.63	1.49, 1.79	−0.14	−0.16, −0.12
Occupation						
Farmer	1	N/A	1	N/A	0	N/A
Factory worker	1.20	1.11, 1.30	1.13	1.07, 1.19	0.001	−0.01, 0.01
Ethnicity						
Han	1	N/A	1	N/A	0	N/A
Other race	1.83	1.30, 2.58	1.34	1.05, 1.72	−0.16	−0.22, −0.10
Folic acid use						
No	1	N/A	1	N/A	0	N/A
Yes	0.88	0.80, 0.97	0.95	0.89, 1.01	0.03	0.02, 0.05
Preconception anemia						
No	1	N/A	1	N/A	0	N/A
Yes	1.16	1.02, 1.31	1.13	1.07, 1.20	−0.03	−0.04, −0.02

**Table 4:** The associations between LBW, SGA, and BWSDS with preconception hemoglobin (Hb) concentrations of women in China, 1993–1996.

Category of Hb Concentrations (g/L)	No. of Participants	LBW			SGA			BWSDS	
		Incidence %	Crude RR (95% CI)	Adjusted * RR (95% CI)	Incidence %	Crude RR (95% CI)	Adjusted * RR (95% CI)	Mean ± SD	ß (95% CI)
Classification 1									
No anemia	96,942	2.01	1	1	5.48	1	1	0.01 ± 0.99	0
Mild anemia	22,495	2.39	1.19 (1.08, 1.31)	1.17 (1.06, 1.28)	6.33	1.16 (1.10, 1.24)	1.14 (1.07, 1.21)	−0.03 ± 1.02	−0.03 (−0.05, −0.02)
Moderate-to-severe anemia	5288	2.29	1.14 (0.95, 1.37)	1.10 (0.92, 1.33)	6.18	1.14 (1.01, 1.28)	1.34 (0.99, 1.24)	−0.02±1.03	−0.02 (−0.05, 0.01)
Classification 2									
<80	164	3.05	1.67 (0.69, 4.09)	1.61 (0.66, 3.95)	7.32	1.50 (0.83, 2.71)	1.46 (0.81, 2.63)	−0.05 ± 1.08	−0.07 (−0.22, −0.88)
80–109	27,619	2.37	1.29 (1.16, 1.44)	1.24 (1.11, 1.38)	6.29	1.28 (1.19, 1.37)	1.23 (1.15, 1.32)	−0.03 ± 1.02	−0.05 (−0.06, −0.03)
110–119	45,570	2.12	1.15 (1.04, 1.28)	1.12 (1.02, 1.24)	5.96	1.21 (1.13, 1.28)	1.17 (1.10, 1.25)	−0.01 ± 1.00	−0.03 (−0.04, −0.01)
120–129	36,114	1.84	1	1	5.00	1	1	0.03 ± 0.98	0
130–139	11,196	1.94	1.05 (0.90, 1.23)	1.05 (0.90, 1.22)	5.04	1.01 (0.92, 1.11)	1.00 (0.91, 1.11)	0.02 ± 0.97	−0.01 (−0.03, 0.01)
≥140	4062	2.49	1.36 (1.10, 1.68)	1.33 (1.08, 1.65)	5.69	1.15 (1.00, 1.32)	1.13 (0.98, 1.30)	0.01 ± 1.05	−0.01 (−0.05, 0.02)

* Adjusted for age, BMI, education, occupation, ethnicity, and folic acid use.

## Data Availability

The data are available in the main text, or can be obtained by contacting the corresponding author (Nan Li).
